# Lessons Learned from Developing Digital Teaching Modules for Medical Student Education in Neurosurgery during the COVID-19 Pandemic

**DOI:** 10.3390/healthcare9091141

**Published:** 2021-09-01

**Authors:** Rosita Rupa, Mirza Pojskic, Christopher Nimsky, Benjamin Voellger

**Affiliations:** Department of Neurosurgery, University Hospital Marburg, Baldingerstr., 35033 Marburg, Germany; rupar@med.uni-marburg.de (R.R.); mirza.pojskic@uk-gm.de (M.P.); nimsky@med.uni-marburg.de (C.N.)

**Keywords:** COVID-19, digital teaching modules, feedback, learning management system, medical student education, neurosurgery

## Abstract

Background: The coronavirus 2019 (COVID-19) pandemic forced students and teachers to rapidly adopt digital education methods. Proper guidance for and refinement of such methods is continuously required. Here, we report on the educational experience students and academic staff at the neurosurgical department of a German university hospital made with digital teaching modules (DTMs) that were newly developed due to the transition to digital teaching during the first year of the COVID-19 pandemic and on the insights gained therefrom. Methods: Nine newly created DTMs provided students the option to anonymously evaluate each module by assigning a score from 0 (worst value) to 5 (best value) to it. Access count, evaluation count, average evaluation, number of included (interactive) figures, number of presented cases, number of linked publications, and number of included multiple-choice questions for each DTM were recorded retrospectively. For each DTM, we aimed to correlate access count, evaluation count, and average evaluation with the number of included (interactive) figures, number of presented cases, number of linked publications, and number of included multiple-choice questions. E-mail responses from individual students as to the DTMs were collected. Among students, an anonymous, voluntary online survey regarding the DTMs was conducted. Results: Number of figures and average evaluation per DTM were significantly positively correlated (Spearman’s *rho* = 0.85; *p* = 0.0037). Number of figures and number of evaluations per DTM were also significantly positively correlated (Spearman’s *rho* = 0.78; *p* = 0.0137). Responses from individual students indicated that illustrative cases and interactive figures might further increase DTM popularity. Conclusion: As a valuable adjunct in medical student education, DTMs should contain (interactive) figures, illustrative cases, a scoring option, and the option to give individual feedback towards the academic staff.

## 1. Introduction

Coronavirus disease 2019 (COVID-19) is an infectious condition caused by severe acute respiratory syndrome coronavirus type 2 (SARS-CoV-2) [1]. COVID-19 has an incubation period of up to 14 days during which the virus may already be transmitted [1]. For COVID-19, a basic reproduction number (R_0_) of 2.5 has been estimated [1]. COVID-19 case fatality rates (CFR) of approximately 4 per cent have been encountered in some highly developed countries, such as Denmark and Germany, and much higher CFR have been observed elsewhere [2]. In a systematic meta-analysis, the infection fatality rate during the first wave of COVID-19 was found to be 0.68 per cent [3]. Since the end of 2019, COVID-19 has evolved into a pandemic [1] that made governments worldwide implement unprecedented non-pharmacological interventions in order to respond to the rapid spread of this new, life-threatening disease [4]. One of these measures, namely the enforcement of social distancing, resulted in fast transition from traditional teaching to digital teaching where feasible [5,6,7].

Prior to the emergence of COVID-19, we had already had access to a server hosting a derivative of the open-source digital learning management system (LMS) ILIAS (https://www.ilias.de) (accessed on 27 June 2021) [8] tailored for medical education (“Knowledge-Based Medical Education” (k-MED)) (https://www.kmed.uni-giessen.de) (accessed on 27 June 2021) [9] at our university. At that time, medical student education at our neurosurgical department had mainly consisted of traditional lectures and seminars and with daily changing attendees, bedside, and operating room (OR) teaching. In March 2020, the first lockdown in Germany (https://www.bundesregierung.de/breg-de/themen/coronavirus/beschluss-zu-corona-1730292) (accessed on 27 June 2021) [10] prompted us to implement contact tracing and social distancing measures, including digital teaching and changed schedules for approximately 160 students per term: lectures were thenceforth made available as moving picture experts group 4 (.mp4) files on the ILIAS server, while bedside and OR teaching necessarily continued with attendees changing weekly or biweekly in order to facilitate contact tracing. For the seminars, a hybrid approach was chosen: the option to attend traditional seminars as long as regulations allowed this (with mandatory enrolment prior to each event in order to facilitate contact tracing) was complemented with 9 ILIAS digital teaching modules (DTMs, Table 1) that authors B.V. and R.R. had developed from scratch after the first lockdown. The content of the DTMs matched the educational aims of the fourth year medical students’ course in neurosurgery at our university. At the beginning of each term, all students—each at the same level of experience—were provided with a catalogue of the educational aims. Each DTM was designed to be followed independently from other DTMs, with prerequisites, i.e., recommended book chapters or video lectures, clearly defined. Numbers of figures, interactive figures (i.e., figures with additional information, such as the names of anatomical landmarks revealed by a hovering mouse pointer), cases, linked publications, and multiple-choice questions varied between DTMs (Table 1).

Each DTM contained the e-mail address of an academic staff member (author B.V. or R.R.) responsible for collecting individual feedback from students and for clarifying any questions regarding the content of the DTM. In addition, students were given the opportunity to discuss any open issues during two online revision courses held by author B.V. at the end of the winter term 2020/2021.

During the COVID-19 pandemic, educational institutions worldwide have faced a similar need for transition to digital teaching [5,6,7], while students, particularly in low- or middle-income countries, may encounter new or increasing barriers complicating educational access [11,12,13,14]. Maity et al. [14] reported that university students, probably due to their overall higher maturity and commitment, appear to be less affected by the difficulties that arise from the pandemic-driven transition to digital education than students at school or at college. In their analysis of data on user experience collected among students after the onset of the pandemic, Chen et al. [15] found that the quality of the technical framework and of the content as well as the design of the user interface determine students’ satisfaction with online education platforms. In 2021, Ramos et al. [16] published a review on works from the pre-COVID-19 era assessing video-based learning (VBL) methods. They conclude that VBL may improve students’ and teachers’ educational experience when going beyond one-way video lecturing [16]. Suggested methods include collaborative VBL, collaborative video analysis, collaborative video authoring, and the use of video annotation tools [16]. Katz et al. [17] recently reviewed numerous examples of how social media platforms (SMPs) were successfully deployed for the transfer of medical knowledge to professionals, students, and patients. With SMPs, however, caution must be taken to avoid the spread of misinformation [17].

As a consequence, digital literacy is, more than ever, considered a prerequisite for students and teachers [12,14,17]. Future publications in the field of digital education are expected to provide guidance for, to evaluate, and to refine recently adopted methods [17,18]. Here, we would like to report on the educational experience students and academic staff at our neurosurgical department made with the newly developed DTMs during the first year of the COVID-19 pandemic and on the insights we have gained therefrom.

## 2. Materials and Methods

Each student had the option to anonymously evaluate each DTM by assigning a score of 0 (worst value) to 5 (best value) to it. We retrospectively recorded access count, evaluation count, average evaluation, number of included (interactive) figures, number of presented cases, number of linked publications, and number of included multiple-choice questions for each DTM on 6 March 2021 (Table 1). For each DTM, we aimed to correlate access count, evaluation count, and average evaluation with the number of included (interactive) figures, number of presented cases, number of linked publications, and number of included multiple-choice questions (Table 1 and Table 2).

Until 6 March 2021, e-mail responses from individual students regarding the DTMs were collected by authors B.V. and R.R. From 8 February 2021 to 1 March 2021, we conducted among our students an anonymous, voluntary online survey as to the DTMs (Supplementary Table S1). We placed the survey at the top of our DTM list and advertised the survey through our ILIAS weblog.

Statistical analysis was conducted, and figures were created using RStudio version 1.3.959 running R version 4.0.2 (https://www.r-project.org) (accessed on 30 June 2021) [19] on a Mac OS X 10.14.6. Spearman’s rank correlation coefficient, *rho*, was estimated. Statistical significance was assumed with *p* values less than 0.05.

## 3. Results

**Figure 1:**
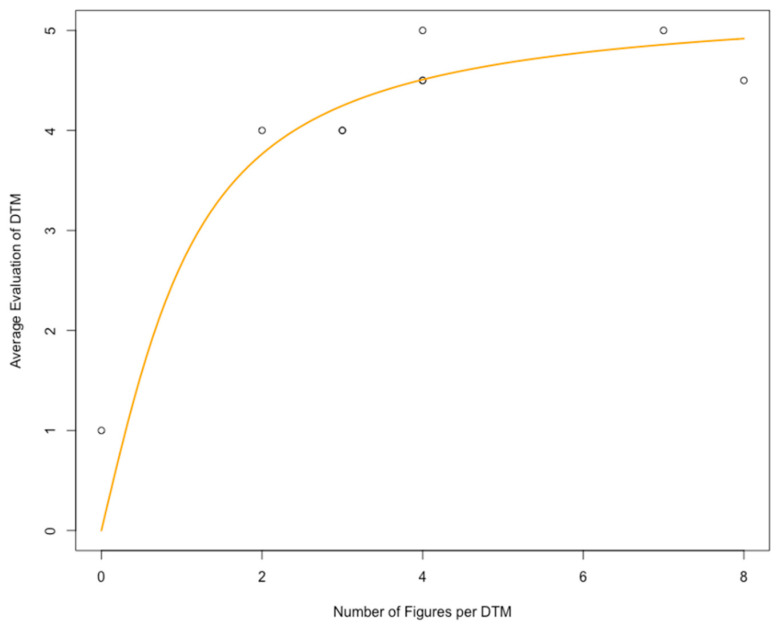
Number of figures per digital teaching module (DTM) and average evaluation (0: worst value; 5: best value) of the respective module.

Up to 13 scores per DTM were obtained (Table 1). A significant positive correlation was found between the number of figures included in a DTM and the average evaluation of the DTM (Spearman’s *rho* = 0.85; *p* = 0.0037; Table 2; Figure 1).

An arc tangent curve (Figure 1) was manually fitted to the data representing the number of figures (x) and the average evaluation per DTM (y) by author B.V. as follows:

y = 3.4 * arctan (x)

**Figure 2:**
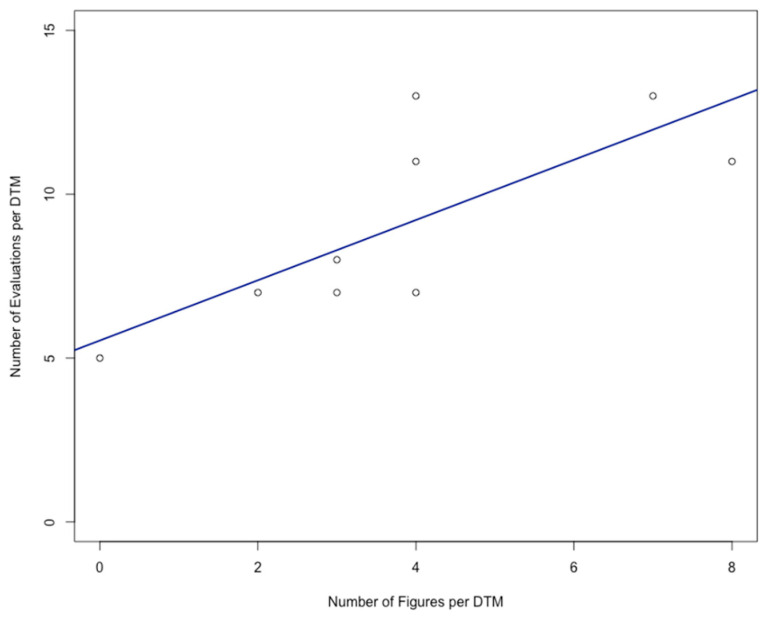
Number of figures per digital teaching module (DTM) and average number of evaluations of the respective module.

A significant positive correlation was also found between the number of figures included in a DTM and the number of evaluations per DTM (Spearman’s *rho* = 0.78; *p* = 0.0137; Table 2; Figure 2). Using the “lm” function in R, a regression line (Figure 2) was automatically fitted to the data representing the number of figures (x) and the number of evaluations per DTM (y) as follows:

y = 5.5355 + 0.9194 * x

One student answered solely to the open question in our online survey, indicating agreement with e-mail responses that we had received from two individual students stating that illustrative cases and interactive figures were found particularly helpful in the transfer of knowledge through DTMs. Eight students responded to the closed questions (Supplementary Table S1) but skipped the open question in our online survey.

## 4. Discussion

There is an abundance of literature on feedback mechanisms in traditional medical student education. Comprehensive reviews on this topic have been provided by Keifenheim et al. and by Lerchenfeldt et al. [20,21]. Such feedback mechanisms, however, are based upon the principles of direct, hurdle-free verbal and non-verbal communication between two or more individuals. By contrast, video conferences, lecture videos, DTMs, and all other digital education channels contain a more or less permeable barrier to the direct interaction between participants [22].

While there is reason to believe that digital teaching will never replace bedside teaching [23], medical student education during the pandemic may certainly become more interesting through pre-made DTMs or similar digital collections of clinical case presentations, such as Capsule (https://www.capsule.ac.uk) (accessed on 27 June 2021) [24,25] or Eurorad (https://www.eurorad.org) (accessed on 27 June 2021) [26]. It remains the responsibility of the teacher to develop DTMs of high quality and to make the content of digital lessons as attractive and comprehensible as possible for medical students, and there is a demand for further research on this topic [23,27]. We believe that carefully designed DTMs will retain their place in medical student education after the COVID-19 pandemic. Beyond that, patients may benefit from easy-to-follow digital medical education tools provided by their doctors during the pandemic and thereafter [28].

The use of digital LMSs, such as ILIAS for medical student education, has been described by various authors and even more so since the emergence of COVID-19 (https://pubmed.ncbi.nlm.nih.gov/?term=digital+learning+management+system) (accessed on 27 June 2021) [29]. There is a wealth of ideas to promote the transition to digital teaching, including coverage of feedback mechanisms that are directed towards the students in settings similar to the situation at our department [30,31,32,33,34]. However, only little light has been shed upon the options digital medical student education provides for feedback that is directed towards the academic staff [31,33,34,35].

In our hands, the exploitation of the digital LMS feedback option with the lowest possible threshold, namely the retrospective analysis of students’ overall evaluation of DTMs with scores from 0 to 5, yielded novel, significant insights (Figure 1 and Figure 2; Table 3): first, the average evaluation of a DTM was significantly correlated with the number of figures provided in the DTM, obviously following a saturation curve (Figure 1). Second, the number of evaluations of a DTM was significantly correlated with the number of figures provided in the DTM in a more or less linear manner (Figure 2).

The number of responses to our extra advertised, prominently placed online survey was disappointing. We assume that this low response rate is primarily owed to the high threshold that came with the survey, namely the considerable amount of time needed to answer all the questions. This part of our experience is similar to what Vielsmeier et al. [34] encountered at the ear, nose, and throat (ENT) department of a German university hospital: despite repetitive requests, there was a low willingness among students to give feedback through an online survey on the quality of digital education during the COVID-19 pandemic.

Such observations may explain why there is little literature on feedback towards the academic staff in digital medical education. The low overall response rate, the retrospective character of our analysis, and the fact that we report on a single-center experience are certainly the main limitations of our study. Nonetheless, we think that the response to the closed questions in our online survey contains a clear mandate to develop more DTMs in the future (Supplementary Table S1).

Although certainly lowest in number and, due to an approach that was qualitative in nature, the single response to the open question in our survey and the e-mail notifications as obtained from individual students brought some additional design advice: these respondents found interactive figures and clinical cases in the DTMs very helpful (Table 3).

## 5. Conclusions

Based on our pandemic-driven experience, we devised the following recommendations for the design of DTMs in medical student education:

- (Interactive) figures and illustrative cases apparently foster students’ engagement with the content of the DTM; and
- To obtain valuable feedback from students, it is advised to offer low-threshold communication channels. These include a scoring option for each DTM, open survey questions, and provision of the e-mail address of a correspondent academic staff member.

## Figures and Tables

**Table 1:** Characteristics of 9 digital teaching modules.

Topic	Average Evaluation	Number of Evaluations	Number of Accesses	Number of Figures	Number of Inter-Active Figures *	Number of Questions	Number of Cases	Number of Linked Papers
Aneurysmal Subarachnoid Hemorrhage	4.5	13	90	4	0	2	1	1
Impairment of Consciousness	4.5	11	162	4	0	5	1	2
Elective Neurosurgery During the COVID-19 Pandemic	4.0	7	77	3	0	2	1	1
Fluorescence-Guided Glioma Surgery	1.0	5	85	0 **	0	2	1	3
Intracranial Pressure	5.0	13	98	7	0	6	1	0
Brain Death	4.0	7	126	2	0	4	0	2
Hydrocephalus	4.5	11	134	8	3	1	1	0
Lumbar Disc Hernia	4.0	8	109	3	0	4	2	0
Cervical Disc Hernia	5.0	7	91	4	1	2	1	0

* Subset of the number of figures; ** the papers linked to this module contained several figures.

**Table 2:** Correlation between design and popularity of 9 digital teaching modules.

Module Popularity Measure	Number of Figures per Module	Number of Interactive Figures per Module	Number of Cases per Module	Number of Questions per Module	Number of Linked Papers per Module
Number of accesses per module	0.4 (0.286)	0.25 (0.5147)	−0.09 (0.8153)	0.34 (0.3692)	−0.13 (0.7354)
Number of evaluations per module	0.78 (0.0137) *	−0.01 (0.9762)	0.19 (0.6294)	0.29 (0.4465)	−0.43 (0.2449)
Average evaluation per module	0.85 (0.0037) *	0.45 (0.2232)	0 (1)	0.17 (0.6653)	−0.62 (0.0743)

Spearman’s *rho* served as statistical test. Data are presented as *rho* (*p* value). * Statistically significant.

**Table 3:** Characteristics of feedback as received through different feedback channels.

Feedback Channel	Threshold	Feedback Quantity	Feedback Quality
Scoring bar	(+)	(+++)	(+++)
Survey, closed questions	(+++)	(++)	(+)
Survey, open questions	(++)	(+)	(++)
Individual response by e-mail	(++)	(+)	(++)

(+) low, (++) medium, (+++) high.

## Data Availability

All data can be obtained from the corresponding author upon reasonable request.

## Supplementary Materials

The following are available online at https://www.mdpi.com/article/10.3390/healthcare9091141/s1, Table S1: Students’ answers to the closed questions in an online survey assessing 9 digital teaching modules.
