# Supplementary material for: Lessons Learned from Developing Digital Teaching Modules for Medical Student Education in Neurosurgery during the COVID-19 Pandemic
Source: Healthcare (Basel). 2021 Sep 1;9(9):1141. doi: 10.3390/healthcare9091141 (PMC8471382; doi:10.3390/healthcare9091141)
Supplement: Supplementary file 1 [file healthcare-09-01141-s001.zip › Table_S1.pdf]

Rupa, R.; Pojskic, M.; et al.: Table S1. Students' answers to the closed questions in an online survey assessing 9 digital teaching modules.

Question / Statement	Possible answers	Most frequent answer
The content of each module matches the topic.	• Absolutely true<br>• True<br>• Less true<br>• Not true	Absolutely true
The required knowledge of each module is clearly defined.	• Absolutely true<br>• True<br>• Less true<br>• Not true	True
The learning objective of each module is clearly defined.	• Absolutely true<br>• True<br>• Less true<br>• Not true	True
The common thread of each module is recognizeable.	• Absolutely true<br>• True<br>• Less true<br>• Not true	Absolutely true
Please rate how demanding the modules are, measured by your previous level of knowledge.	• The modules are much too demanding.<br>• The modules are demanding.<br>• The modules have exactly the right level.<br>• The modules are not very demanding.<br>• The modules are far too little demanding.	The modules have exactly the right level.
Theoretical and practical aspects of each module are coordinated.	• Absolutely true<br>• True<br>• Less true<br>• Not true	True
Current scientific discourses and findings are taken into account.	• Absolutely true<br>• True<br>• Less true<br>• Not true	True
I feel to have achieved my learning objectives.	• Absolutely true<br>• True<br>• Less true<br>• Not true	True
Please let us know your opinion as to the number of the modules.	• I would like to see significantly more modules in the future.<br>• I would like to see more modules in the future.<br>• The number of modules is just right.<br>• I would like to see less modules in the future.<br>• I would like to see significantly less modules in the future.	I would like to see more modules in the future.
I mainly access the teaching materials through:	• A private computer at home.<br>• A private computer at the university.<br>• A public computer at the university.<br>• A public computer elsewhere.<br>• Another device.	A private computer at home.

Rupa, R.; Pojskic, M.; et al.: Table S1 (continued). Students' answers to the closed questions in an online survey assessing 9 digital teaching modules.

The graphics are clear.	• Absolutely true<br>• True<br>• Less true<br>• Not true	Absolutely true
The text is easy to read.	• Absolutely true<br>• True<br>• Less true<br>• Not true	Absolutely true
The media used are appropriate.	• Absolutely true<br>• True<br>• Less true<br>• Not true	Absolutely true
In comparison to classical seminar lessons, the modules offer:	• Less incentive to gain knowledge.<br>• A similar incentive to gain knowledge.<br>• A higher incentive to gain knowledge.	A similar incentive to gain knowledge.
In comparsion to classical seminar lessons, the modules convey the learning content:	• Less clear.<br>• Similarly clear.<br>• Clearer.	Similarly clear.
